# The edible insect sector in Canada and the United States

**DOI:** 10.1093/af/vfad047

**Published:** 2023-08-14

**Authors:** Jennifer Larouche, Barbara Campbell, Louise Hénault-Éthier, Ian J Banks, Jeffery K Tomberlin, Cheryl Preyer, Marie-Hélène Deschamps, Grant W Vandenberg

**Affiliations:** Ribozome, Québec City, QC, Canada; Natural Products Canada, Charlottetown, PEI, Canada; Institute National de la Recherche Scientifique, Québec City, QC, Canada; Enviroflight, Maysville, KY, USA; Texas A&M University, Department of Entomology, College Station, TX, USA; Center for Environmental Sustainability through Insect Farming, Texas A&M University, College Station, TX, USA; Université Laval Faculté des sciences de l'agriculture et de l'alimentation, Département des sciences animales, Québec City, QC, Canada; Université Laval Faculté des sciences de l'agriculture et de l'alimentation, Département des sciences animales, Québec City, QC, Canada

**Keywords:** animal farming policies and regulations, entomophagy, frass, mass insect production, sustainable protein

**Implications**

- Edible insect markets are rapidly expanding, driven by consumer demand for sustainable food.
- Edible insect production volumes are still too small to drive a massive change in animal feed sustainability.
- Frass regulations may delay the development of the nascent edible insect market if they are not simplified and harmonized with other agricultural waste management practices such as manure.
- Information for the general public about the insects in the food and feed industry in Canada and the United States is lacking and should be addressed by supporting organizations with the collaboration of the industry.

## Introduction

The history of edible insects in North America (NA) dates back to the precolonisation era when indigenous knowledge about insect consumption may have saved early settlers (Schrader et al., 2016). In the United States, early insect-farming activities were related to fishing baits (crickets) (Hall et al., 2021) and the oldest known enterprise, Armstrong Cricket Farm, founded in 1947, is still active (Wilkie, 2018). Other large-scale insect-culture activities in this region focused on the production of species used in biological control of pests (or integrated pest management) within the forestry and agri-food sectors or more recently to control insect populations carrying transmissible pathogens that threaten humans and other vertebrates (Klassen and Vreysen, 2021). The relatively recent interest in large-scale production of edible insects has closely mirrored that observed in Western European countries. The 2013 report by the FAO (van Huis et al., 2013) and related efforts laid the foundation for significant developments that have taken place since; both academic and industrial progress has been unprecedented to permit the emergence of large-scale culture of a range of insect species as food and feed (NPC, 2022). The underlying factors motivating the emergence of this sector shares several commonalities with those in Europe, including increased consumer demand for eco-responsible ingredients such as food and feed (Hénault-Ethier et al., 2020) paralleled by matching marketing approaches (Marquis et al., 2020), concerns of negative environmental impacts of traditional food and feed production, and increased pressure in many jurisdictions to manage food waste in a circular manner (Hénault-Ethier et al., 2017; Lahteenmäki-Uutela et al., 2017).

The aim of this article is to review the current state of the art related to edible insects in Canada and the United States, including production sectors, regulatory frameworks and R&D efforts to provide an updated status, opportunities, and challenges faced by the NA edible insect sector.

## Commercial Concerns

Insect farms generate coproducts, insects and frass, for which the final market will be depending on the insect species and the processing methods used. For most production, insects are further processed into full meals, defatted meals and oil which are sold for human and animal consumption. While several studies are proposing protein extraction methods (Ravi et al., 2020), no producers in Quebec are offering this product yet (TFIC, 2022) and minimal information is publicly available about the products being ready to reach the market.

Specialists forecast market receptivity and growth both in NA and Europe (Mancini et al., 2022). Although typical profiles of insect consumers are emerging in NA (Marquis et al., 2020), lack of market knowledge, production inconsistencies, and low production volumes represent challenges for the commercial success of edible insects. As for any new industry, the number of active companies and production volumes is still partially defined or kept confidential (Dussault, 2017). Over the last few years, the market interest is reflected by a growing number of market reports related to edible insect proteins. Although all agree on the potential of this emerging sector, the conclusions of these reports are highly variable (Mancini et al., 2022) with some more confident than others.

**Figure 1.**
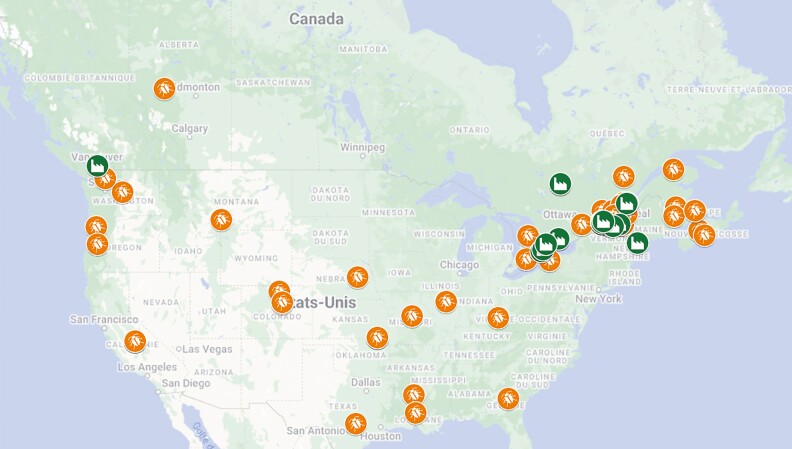
Map of edible insect producers (orange beetle, excluding producers of live insects for the pet market and research centers) and processors (green facility) in Canada and the United States.

The sector is now gaining momentum with several primary insect producers across the continent and value chain partners downstream beginning to incorporate insects as a primary ingredient in their products. The number of active insect-based companies is hard to establish considering the high turnover of start-ups (opening and winding down). Studying a precise geographic location (province of Quebec, Canada), the rapid growth of the industry has been observed since 2015 with an average annual growth in the number of companies of 29% (TFIC, 2022). But defining the growth over the larger NA region is challenging because there is no organization officially responsible for keeping track of the industry growth. By combining the information from the edible Quebec Insect Sectorial Table (TFIC), Natural Product Canada (NPC) and the North American Coalition for Insect Agriculture (NACIA) members, it was possible to obtain a rough estimate of 41 insect producers in Canada and 21 in the United States in 2022 (NPC, 2022; TFIC, 2022). In addition, there were 22 companies offering insect-based products in Canada and one in the United States (Figure 1). A better knowledge of the number of active companies is required to better evaluate the growth of the industry.

As with any new industry sector, NA insect agriculture has faced challenges of consistency, with highly varied methods of production and downstream processing being applied to the three major industrial species. Harmonization efforts are underway, including an industry-led initiative through the NACIA to adapt the International Platform of Insects for Food and Feed (IPIFF) best practices guideline for NA producers. International efforts to harmonize feed assay protocols for black soldier flies (Bosch et al., 2019) and mealworms (Deruytter et al., 2022), including NA research groups and industries, are also deployed to help accelerate the optimization of insect nutrition. Increasing consistency of production should help engage early adopters, relying on insect protein and oils that meet their certificates of conformity. But increasing consistency may also help the second major challenge of the industry—production scale. The market pull for alternate proteins and healthy oils vastly outstrips the current global insect production to such a degree that currently no individual insect farm alone, nor all insect farms combine, for a given species could meet the scale needed for the successful commercialization of an insect-based aquaculture feed, pet food or poultry feed. With more consistent production from multiple insect farms, the outputs can be pooled and begin to fuel the production of downstream insect-based products.

The appetite for sustainable, nutrient-rich, high-quality protein and oils from insects has already been demonstrated in aquaculture, pet food, and animal feed. In fact, the barrier to widespread market penetration is scale. In Canada for example, the minimum tonnage for commercial launch of a national dog food with insect protein is 10 tonnes. A scale hardly met by any individual Canadian farm to date. According to a market study lead by Enterra, the demand for high-quality insect ingredients and products was 150,000 T in NA and Europe in 2020 and is expected to increase with a high growth rate (Jowett, 2020) which needs to be estimated. In 2019, European production was estimated at 500 T and is expected to expand to 260,000 T by 2030 (Grau et al., 2022; Mancini et al., 2022). Therefore, considering the advance of the European production volume, the immediate demand for insect products was not reached when combining both productions.

**Figure 2.**
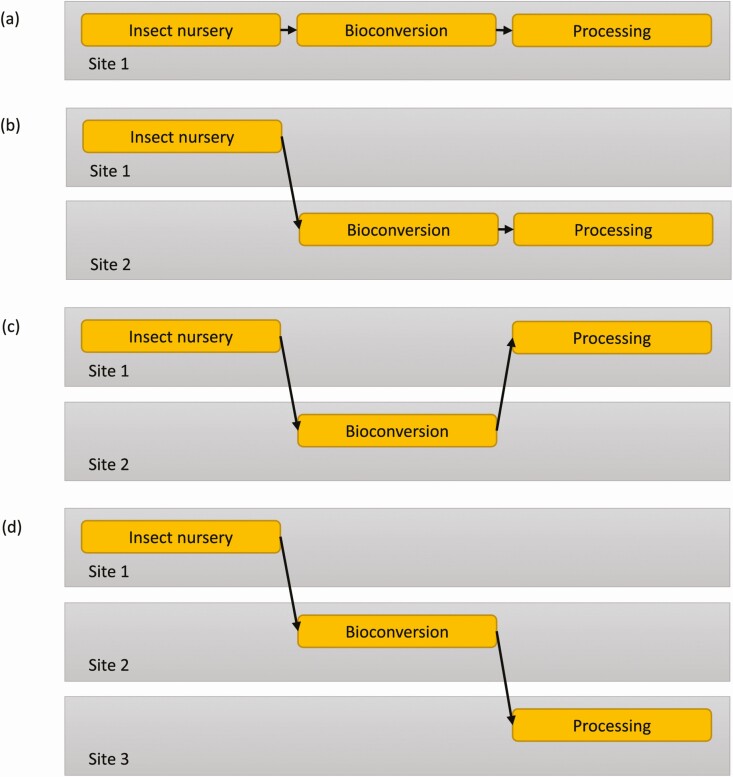
Market expansion strategies: (a) centralized and integrated plan, (b) centralized nursery with decentralized bioconversion and processing, (c) centralized nursery and processing with decentralized bioconversion and (d) decentralized plan. Adapted from figures in Grau et al. (2022).

Two market expansion strategies are unfolding in NA (Figure 2). First, there is a “gold rush” to increase the production volumes of individual industrial players and secure market shares while reducing product prices. This strategy is the most common, but it represents the highest risk with important capital requirements while the market is slowly expanding. In 2023 Aspire Food Group’s “world’s largest” cricket farm should reach full capacity and Entosystem brings their 100,000 sq ft black soldier fly expansion facility online, but significant growth is needed across the sector to meet and then sustain market penetration of insect protein and oil. In the United States, several companies have undergone significant development, such as Enviroflight, InnovaFeed, Beta Hatch, PreZero, and soon, Ynsect and Protix will help address capacity issues there. The second strategy for edible insect sectorial growth involves tight cooperation between smaller specialized (decentralization) enterprises (nursery, bioconversion, and processing) deployed over a wider geographical area (Grau et al., 2022), similar to the conventional poultry and pig farming industry. This approach has been demonstrated in Europe and is slowly gaining traction in NA. If each market (dog food, aquaculture feed, and poultry feed, etc.) requires a minimum of 100,000 tonnes to fulfill the market need in NA, then even global production, let alone NA production, will continue to fall short for the next 5–10 yr unless efforts are made to sustain sectorial growth as a whole. Much hinges on the success of these recent expansions, as they are per if they are proven commercially viable and the combination of protein, oil and frass sales provides them with profitability, then their traction should pave the path for additional production capacity.

## Regulations Applied to Insect

The edible insect industry is generating several coproducts that can be redirected toward five main markets, human food, pet food, feed, fertilizer, and other applications, which include pharmaceutical and cosmetics. Both in Canada and the United States, there are federal requirements that supersede provincial or state legislatures in the food, feed, and fertilizer sectors which can sometimes make regulations that pertain to insects difficult to navigate. Historic regulations on insects generally regarded them as filth and potential agents of adulteration in food. Insect-specific regulations are still lacking making the regulatory scenery uncertain for market expansion.

### Food

Canada’s regulations consider that if there is a documented history of traditional consumption of a food item elsewhere on the globe, it can be sold here without further regulatory constraints, but must first undergo the novelty determination process. Because of this, several insect species are already considered as nonnovel food, including, but not exclusively, the mealworm (*Tenebrio molitor*), the lesser mealworm (*Alphitobius diaperinus*) and the banded cricket (*Gryllodes sigillatus*) (Government of Canada, 2022a). However, every insect species which is not approved on the *List of nonnovel determinations for food and food ingredients* (Government of Canada, 2022a), such as grasshoppers or black soldier fly, require a novel food determination during which companies must demonstrate the history of safe consumption. However, if the insect species was considered a novel food by Health-Canada during the novelty determination process, a complete demonstration of the product’s nutritional, chemical, microbiological and toxicological safety before going to market (Government of Canada, 2022b). Beyond this federal requirement, each province is responsible for agricultural production standards and food safety guidelines (Lahteenmäki-Uutela et al., 2017).

The U.S. regulatory system, the Food and Drug Administration (FDA), controls insects as food under the Federal Food, Drug, and Cosmetic Act. As with other foods, edible insects could be Generally Recognized as Safe (GRAS) if a firm or a third party demonstrated relevant scientific opinion based on published or unpublished scientific data (Lahteenmäki-Uutela et al., 2017). But these expensive testing procedures are prohibited in a nascent industry. Else, edible insects may be considered food additives as they may reasonably become components or otherwise affect the characteristics of food, and as such, it requires premarket review and approval by the FDA. While specific mandates on insects as food are still lacking, the FDA has expressed written opinions on the fact that insects may be considered food if that is their intended use and if they follow the regulatory requirements of other foods. These requirements involve being clean and wholesome, produced and otherwise processed under sanitary conditions following good manufacturing practices (GMP), and properly labeled. Insects farmed for animal consumption or collected in the wild may not be diverted to the food market. Because regulations are still ill-adapted to insects as food, each company markets it at its own risk. Obtaining GRAS or FDA food additive supplement recognition is required for every individual insect species which could overwhelm the agency’s capacity.

### Pet food and treats

Only a few government restrictions apply in Canada regarding pet food and treats, but insect ingredients must be produced in sanitary conditions (TFIC, 2022) and distributors require producers to follow Good Manufacturing Practices (GMP) and an array of safety data for pet food ingredients. In the United States, pet food is under the nongovernment Association of American Feed Control Officials (AAFCO). In 2022, AAFCO approved the dried black soldier fly larvae ingredient as safe for adult dogs which unlocked the potential for a large market in the United States (AAFCO, 2021). However, approvals must still be obtained for gestating per lactating female dogs, puppies, and all three life stages in cats. Furthermore, insect producers working with different insect species must go through the approval process for their feed ingredients in all dog and cat life stages.

### Animal feed and functional claims

In the United States, the key organization for developing regulations associated with animal feed is AAFCO. This independent body, which is comprised of individual state control officials and governmental employees volunteering their time, provides guidelines for the sale and distribution of animal feed, including pet food, across geopolitical scales ranging from local to federal; however, the approval of regulations is managed by the FDA and state authorities. In most instances, entities seeking regulatory guidance are recommended to communicate with the AAFCO; however, engaging FDA is permissible. With that said, determining whom to contact is challenging simply in part, as pointed out initially, to the complexity of the FDA.

Further complications arise in the marketing and sale of animal feed ingredients that have functional benefits. The current FDA regulations and guidelines mean that the labeling claims for animal foods are limited to the effects of the ingredients’ nutritional properties. This framework means that if a company wants to make functional claims, such as benefits to the environment, improved growth or reproductive behaviour, improved milk per meat per egg production, or improved physiological conditions, the product is considered a drug, and requires a separate, and much lengthier and more expensive, approval process. These regulations are seen as outdated by the broader U.S. animal feed industry and are behind many other regions, including the EU, Canada, Brazil, Australia, Chile, and more, and limit innovation, especially in nascent industries such as insect agriculture.

### Fertilizer and plant biostimulants

**Figure 3.**
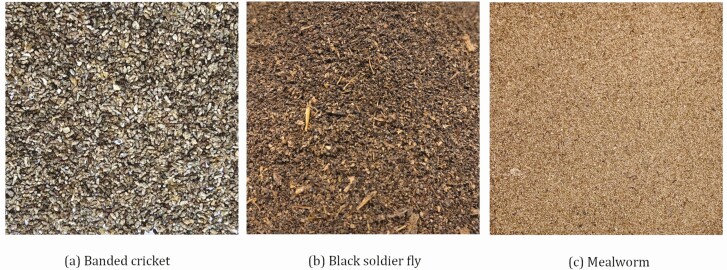
Frass from the three main edible insect species produce in North America are: (a) house cricket, (b) black soldier fly, and (c) mealworm (TFIC, 2022).

Insect frass is probably one of earth’s oldest fertilizers, naturally spread across the landscape by wild insects. Physical attributes/consistency (Figure 3) and N–P–K ratios for insect frass vary greatly across species (Beesigamukama et al., 2022) and production methodologies. Frass is an important coproduct generated during insect production which could be an economic bottleneck to the expansion of the edible insect sector, especially if its definition implies some sort of regulation. The use of coproducts from edible insect production as a fertilizer or soil amendment also depends on federal, provincial or state regulations.

Despite the biological classification of insects as animals, the revamped Canadian Fertilizer Act now excludes insects from the list of animals generating manure. This shift in definition means that the limited sale and export of frass for specified uses currently authorized as “specialty fertilizer” ends as of October 2023, shifting to a requirement for the Canadian Food Inspection Agency (CFIA) for approval as an organic fertilizer and soil supplements (CFIA, 2020). The time and capital required to gain authorization may hamper the edible insect sectorial growth. However, the frass working group from the TFIC considers frass as insect manure, which would alleviate the regulatory hurdles for sale and use. There, frass is defined as “Organic material from the production of insects, obtained following the harvesting of insects, mainly composed of insect droppings and which may contain variable quantities of exuviae, insects, litter, or food refusals.’’ (TFIC, 2022). The sale of frass in bulk (≥50 liters) or its use in fields may require provincial ministerial authorizations delivered by competent environmental extension services. To date, only dry frass is sold in Quebec (TFIC, 2022), though industrial research to produce pelletized frass which is easier to apply, increases liberation time and generates less dust is underway.

United States. Frass as a fertilizer is regulated differently in the United States, depending on whether it is used in conventional or organic farming. For conventional farming applications, frass is regulated by the Department of Agriculture of the state the producer is in and may require a license and product registration. Furthermore, any producer selling frass to a different state is required to obtain a license from that state. However, if the frass is to be used for organic farming, it is regulated at the federal level by the USDA National Organic Program (NOP). While the NOP develops and enforces national standards for organically-produced agricultural products sold in the United States, it does not directly certify businesses or products. Instead, it accredits state governmental and independent third-party certifying organizations, such as the California Department of Agriculture (CDFA) or the Organic Materials Review Institute (OMRI), respectively. Unfortunately, the NOP lacks a definition for frass, which has resulted in inconsistent certification of insect frass products by different certifying organizations. This has a negative impact on the nascent industry, as it restricts the higher-value end-use market of organic farming and can create confusion among end-users.

Further complications arise if a producer would like to make functional claims about their frass product. If a producer intends to market frass as a plant biostimulant—a substance that stimulates natural processes to enhance or benefit nutrient update, nutrient efficiency, tolerance to abiotic stress, or crop quality and yield—it is regulated under the Federal Insecticide, Fungicide, and Rodenticide Act (FIFRA). This means the product must be registered as a pesticide with the U.S. Environmental Protection Agency (EPA), and the equivalent state agency for every state the producer intends to sell the product. Also, the importation or interstate movement of biological control agents is regulated by the USDA Animal and Plant Health Inspection Service (APHIS), therefore, APHIS shares dual jurisdiction with EPA when regulating microbes that help plants fight off pests and pathogens.

Considering the myriad of potential and demonstrated functional benefits of frass (Barragan-Fonseca et al., 2022), the current regulatory framework in the United States is a complex barrier that can prevent producers from realizing the full potential of frass.

## Organic Waste Challenges and Considerations

While insects are inherently more sustainable as an industrial-scale food and feed source than other related systems—lower water usage, vertical farming reducing hectares required; there are elements of their production that can be optimized for energy efficiency and lowered carbon footprint. Some of these are apparent—solar roofs on 9,300 m^2^ insect farms go a long way to reducing the energy requirements of the facility; but others come from upcycled or sustainably sourced insect feed.

Obviously, as a sector focused from its inception on being environmentally driven, there have been significant efforts in exploring waste organics as insect feed. Black soldier fly larvae (BSFL) as voracious consumers have led this charge, with mealworm and cricket facilities still relying primarily on prepared feeds (TFIC, 2022). With upcycled feedstock comes a whole array of challenges—availability per scale (a 9,300 m^2^ BSFL farm requires approximately 90 T of wet-weight organics per day as feed); consistency (seasonal fluctuations in crop waste) and perhaps most challenging: depackaging preconsumer organic waste. In NA, insects that are destined for consumption as a food or as a feed ingredient are limited to preconsumer wastes that have met regulatory approval. For example, in the United States, BSFL intended for animal feed applications can only be reared on “feed grade” materials. While this clearly ensures a level of safety, it does exclude postconsumer organics (“green box household waste”). And while there is a significant volume of preconsumer organic waste available, over 50% of food produced ends up in waste streams (Gooch et al., 2019), this potential insect feedstock is heavily packaged. Depackaging these organics at scale has proven a significant barrier to many insect farms. Typical depackaging technology for organics destined for anaerobic digestors or composting involves mechanical separation and sieving, which leaves large amounts of plastic, glass and cardboard fragments in the material. Thorough depackaging with a combination of robotics, scanners and human QA per QC is prohibitively expensive, therefore, innovation is required to produce a cost-effective solution. For an industry that has been able to lean on farm fresh “waste” produce (unpackaged) and food processing sources such as spent grains from breweries, expansion requires solutions to depackaging that are not yet operational.

Product pricing is also an important consideration for the long-term success of the industry. A 2021 report about the market potential for insect protein estimated the sale price of insect protein would drop from € 3,500 to € 1,500 per metric ton by 2030 (de Jong and Nikolik, 2021). As shown in Table 1, this price reduction is expected to occur as the industry matures and will be needed to keep the product competitive with the wider protein market. These price reductions will need to be supported by reductions in capital and operating costs, which can be achieved by further innovation and research.

**Table 1.** Estimated market size and product price per industry phases (adapted from (de Jong and Nikolik, 2021)

Industry phase and estimated product price (EUR per metric ton)	Estimated market size (metric tons)					
	Total	Pet food	Aquaculture	Poultry layers	Poultry broilers	Piglet
Scale-up phase: € 3,500–5,500	120,000	65,000	20,000	20,000	10,000	5,000
Wider-use period: € 2,500–3,500	200,000	85,000	55,000	30,000	20,000	10,000
Maturity phase: € 1,500–2,500	500,000	150,000	200,000	70,000	50,000	30,000

## Increasing Industry Support

Clearly, numerous funding agencies in Canada and the United States are more attentive to the development of insects in the food and feed sector. However, these funds are primarily geared towards the applied sector (i.e., industry), which limits the number of researchers (e.g., university or within the industry) available to conduct said work. Consequently, efforts to expand the value of such research towards basic sciences are needed. By doing so, the number of researchers engaged could expand which would: 1) diversify research activities and topics; 2) enhance industry expansion, and 3) increase emphasis on quality control, optimization, and value.

The development of trade organizations serves as a platform to accomplish numerous goals. Efforts through groups, such as the NACIA, the Association des Éleveurs et Transformateurs d’Insectes du Québec (AÉTIQ) and the TFIC, as well as professional scientific organizations (e.g., Entomological Society of America, Canadian Entomological Society) allow for identifying key issues (e.g., regulatory, research) needing to be addressed. Furthermore, such entities engage communities either directly or through the media which allows for education and potential acceptance of the concept of insects as food and feed. These efforts are needed as they serve as a feedback loop guiding government representatives attempting to determine where to direct research funds. Fruits of such endeavours are paying dividends as agencies, such as the National Science Foundation (NSF) and the U.S. Department of Agriculture (USDA) are investing more than ever in the insects as food and feed sector. This result is best exemplified by the creation of the NSF Industry per University Cooperative (IUCRC) Center for Environmental Sustainability through Insect Farming (CEIF). The CEIF serves as a bridge between industry and academia in order to: 1) address the most pressing research needs; 2) develop the next generation of researchers, and 3) expand the research community by recruiting faculty that historically have not worked in this sector.

In Quebec, the growing interest in the industry has supported the creation of a Leadership Chair in Edible Insect Production and Processing (CLEIC) at the Department of Animal Science of Université Laval, an opportunity to create a new professor per researcher position. First, of its kind in NA, this partnership with the industry and other stakeholders aims to: 1) create an R&D center of excellence (optimization of production techniques, recovery of municipal organic waste, valorization of livestock waste, the definition of economic & territorial models, diversification of edible species production); 2) develop structuring activities at the provincial (creation of an edible insect industry concertation table in Quebec), national (creation of a pan-Canadian research network of insects as food and feed), and international levels (adapting the IPIFF good practice guide to the NA industry, organizing the conference Insects to Feed the World 2020 and 2022) and, 3) improve education and training opportunities (integration of modules in ongoing courses at the agriculture and food faculty; creation of a new course on edible insects from farm to table; summer course, advanced courses for agricultural workers, training courses for young people). The Pan-Canadian Research Network in the field of insects as food and feed aims to: 1) improve communication and collaboration between the different research and training institutions; 2) plan, organize, and administer projects for the advancement of scientific knowledge; 3) strengthen per optimize innovation support for businesses by promoting collaborative projects; 4) build, promote, and exchange training content at different levels of education and, 5) accelerate the training of highly qualified personnel. Taken together, these efforts should assist the emergence and long-term maintenance of the edible insect production sector in Canada in democratizing knowledge and avoiding its loss during an unfortunate closure of industrial leaders.

## Perspectives and Challenges for Future Development

With so much said, where does the industry go? The answer to such a question can be viewed as intimidating; however, the consensus of those involved with the preparation of this publication would lean more towards optimism due to the diverse opportunities currently known. The more pertinent question would be, how does an industry in its infancy develop an organized global plan that leads to a universal language permitting generated data to be adopted globally, regardless of national variance in regulatory guidelines? Developing clear pipelines allowing for data to be organized and made available is key for any company attempting to become established or to expand and diversify. However, presently, data are typically available via scientific literature that is either open access or not. Basically, a large portion of the information needed to develop propositions for regulatory bodies to consider is not accessible. Furthermore, the lack of standard experiment design protocols inhibits the production of uniform data collection and analysis. However, communication is a two-way street. While industry, along with research, attempts to generate the necessary materials for regulatory expansion, navigating the administrative channels within government agencies can be challenging, particularly for a new sector requiring new and specific guidelines. Simple questions, such as whom you contact or how you contact them, remain a “black box” that is constantly evolving. A potential solution would be expanding regulatory bodies to include panel members from the insects as food and feed sector. By doing so, such an individual can engage in research (e.g., CEIF and CLEIC) or commodity groups (e.g., NACIA, IPIFF, AÉTIQ, and TFIC) as a means for relaying “real time” and accurate information to the industry.

Of course, developing the necessary data for regulatory expansion is predetermined by identifying areas of research that need to be addressed. Presently, the industry is experiencing convergent evolutionary processes and appears to be mirroring the path followed by other industries, such as poultry, aquaculture, and other domestic livestock. Streamlining genetics for predictable output could allow for less variance in terms of production but also identifying key research topics that when addressed, benefit the global industry. Similarly, diversifying research to include disciplines, such as but not limited to, engineering, could lead to efficient practices that minimize the environmental impact.

Over the past decade, a range of NA jurisdictions has developed policies to more effectively manage organic waste (OW). For example, California and Quebec have developed policies to eliminate landfilling of organic residues and reduce resulting methane emissions. These programs promote bioconversion via composting or anaerobic digestion (AD) to produce compost or methane as a source of renewable natural gas. Both approaches require significant infrastructure investments, particularly for AD facilities, and consume significant OW quantities. These downcycling bioprocesses represent a significant threat to the potential supply of OW for the insect sector. Although complex, more contemporary and complementary approaches for OW collection per sorting are required to align emerging bioprocesses and maximize potential OW value.

## Conclusion

The edible insect sector in Canada and in the United States is developing quickly but requires significant work to reach a mature industry state. This maturity will be characterized by high production volume for the three main insect species, consistent insect ingredients and well-documented markets. To reach this state, strong collaborations between industrial associations, research centers and academic organisations are required. This could allow generating of meaningful data that would be used to support government organizations in adapting appropriate policies. In addition, there is a continued requirement for research and development, including increasing access to organic waste materials (depackaging strategy, standardized bioconversion approaches for each insect species). A more integrated communication and collaboration strategy within the sector will increase access to data, reduce the chances of repeating the same mistakes by sharing experiences and increase the industry growth rate while encouraging less common market expansion strategies such as value chain specialisation.

*Conflict of interest statement*. Louise Hénault-Ethier is cofounder and research and development director at TriCycle.

Jennifer Larouche is Chief Scientific Officer and shareholder at Ribozome Inc.

## About the Authors

**Jennifer Larouche, M.Sc.** is the Chief scientific officer for Ribozome, a mealworm production company in Quebec, Canada. She graduated with a master’s in Animal sciences at Laval University in edible insect processing. She then worked as an entotechnology director for the Quebec Agrifood Innovation Center before being recruited by the new Quebec Edible Insects Sector Table in Quebec. She also worked as an entotechnology advisor to support new companies in this emerging industry. Jennifer is really implicated in democratising the use of insects as food and was on the local organization committee for Insects to Feed the World 2022 and was responsible for Eating Insects North, a general public event which allowed more than 12,000 people to taste edible insect food prepared by local chefs.

**Barbara M. Campbell** is a serial health and life sciences entrepreneur. In addition to her past role as CEO of Hammock Facilitation Inc., Barbara cofounded two medical device start-ups OrthoMX Inc. and FIVAMed Inc. In her founder roles and with her portfolio of Hammock clients, Barbara propelled emerging technologies and companies through proof of concept, prototype development, clinical validation and multinational partnerships. Barbara is an alum of Université de Strasbourg, Mount Allison University (Hons BSc. Biochemistry, B.A. French), and Simon Fraser University (M.Sc. Biochemistry and Immunology). Currently promoting sustainable company development as Regional Director Atlantic for Natural Products Canada, Barbara continues to add value to environmentally conscious companies and support the Canadian ecosystem for alternate proteins.

**Louise Hénault-Ethier** is currently an adjunct professor at Institut National de la Recherche Scientifique, director of the research center Centre Eau Terre Environnement and board member of Recyc-Québec and Société Québécoise de phytotechnologie. She is a biologist (B.Sc.: Ecology, Concordia University) and Environmental Sciences specialist (M.Sc.: microbial ecology, organic geochemistry and environmental engineering, Concordia University; Ph.D: Environmental Sciences, Université du Québec à Montréal). Her postdoc (Université Laval) focused on entotechnologies: the use of insects to upcycle organic waste. She is cofounder and director of research and development at TriCycle, a circular urban mealworm farm. She has worked as a senior science project manager at the David Suzuki Foundation. Her research interests focus on nature-based technologies using insects, plants or microbiota, urban and regenerative agriculture, organic waste management and nutrient cycles, sustainable fertilizers and pesticide substitutes, carbon footprint, environmental, and health impacts. She employs transdisciplinary methodologies in social and political sciences.

**Ian J. Banks** holds the position of Director of Regulatory Affairs at EnviroFlight and is responsible for directing external and internal projects that help advance North American insect-agriculture regulations. He works closely with external collaborators to develop industry-wide regulatory strategies and industry-specific best practices. Ian is passionate about scaling up insect agriculture as a solution to various environmental and sustainability challenges. Ian has worked on using BSF technology to valorize various waste streams since 2010, in both academia and industry. He has a PhD in Entomology and Sanitation and an MSc in Medical Entomology from the London School of Hygiene and Tropical Medicine, and a BSc in Parasitology from the University of Aberdeen. Ian previously worked for AgriProtein, an innovator in upcycling food waste into insect-derived products, where he helped commercialize large-scale BSF technology by conducting foundational research, product development, project management, fundraising, public relations, and business development.

**Dr. Jeffery K. Tomberlin** is a Professor, AgriLife Research Fellow, & Presidential Impact Fellow in the Department of Entomology at Texas A&M University. He is the principal investigator of the Forensic Laboratory for Investigative Entomological Sciences (F.L.I.E.S.) Facility (https://forensicentomology.tamu.edu) at Texas A&M University, and he is the Director of the National Science Foundation Center for Environmental Sustainability through Insect Farming. His research efforts for the past 27 years have been developing methods to produce alternate protein sources for use as livestock, poultry, and aquaculture feed from these resources. Predominately, these efforts have been accomplished through his research with the black soldier fly. To date, he has edited 8 books, published 28 book chapters, and +200 research articles which have been cited more than 15,500 times. Through his efforts, he was recently recognized as a Fellow by the Entomological Society of America.

**Cheryl Preyer** is the Industry Liaison Officer for the Center for Environmental Sustainability through Insect Farming (CEIF), an NSF-supported Industry-University Cooperative Research Center. CEIF brings together Academia and Industry to identify and perform foundational, precompetitive research supporting the Insect Agriculture sector. After decades in Quick Service Restaurant (Fast Food) Marketing, Cheryl Preyer made the move from fries to flies! With a dawning realization of the large amounts of food wasted in the United States and the need to feed a drastically growing global population, she found herself drawn to insects as a solution. She joined EnviroFlight where she handled business development and marketing communications. She worked for EnviroFlight at their pilot plant through acquisition by Intrexon. Elected to the inaugural NACIA (North American Coalition for Insect Agriculture) board of directions where she served as Board President.

As a biologist specialized in ecology, physiology and animal nutrition, **Marie-Hélène Deschamps** works to find innovative solutions in animal production. Since 2015, she is particularly interested in the valorization of residual organic matter and the mass production of edible insects in order to redirect them to animal feed. Professor Deschamp’s work aims to acquire and transfer new knowledge in this emerging field; create a stimulating research environment to address the various issues of the sector and, acting as a structuring actor of the sector, a general effort to implement an applied model of circular economy and optimize the exploitation of agri-food resources in Quebec.

**Dr. Grant Vandenberg** has a background in animal physiology and aquaculture. He attended the University of Guelph, receiving his BSc in animal science and an MSc in fish physiology and aquaculture. He gained academic experience in the United States, followed by work in the private biotechnology sector He completed his PhD at Laval University working on polymer encapsulation strategies and fish nutrition. Dr. Vandenberg was awarded an NSERC Industrial Chair for the development of Sustainable Aquaculture at Université Laval's Dep. of Animal Science to develop nutritional approaches to minimize the environmental impact of fish production, as well as technological developments related to aquatic animal health, production and physiology. Since 2015 his laboratory has been working to develop the use of insect larvae to upcycle residual organic matter as an alternative approach to manage organic waste, and to develop innovative and high-value ingredients for the animal feed sector.
